# Real-world treatment outcomes in pregnancy and lactation-associated osteoporosis: a nationwide multicenter cohort study

**DOI:** 10.3389/fendo.2026.1934166

**Published:** 2026-09-17

**Authors:** Mehmet Sözen, Zeynep Cantürk, Özen Öz Gül, Mustafa Sait Gönen, Hande Mefkure Özkaya, Serhat Uysal, Esra Şüheda Hatipoğlu, Rukiye Dilara Tekin Uzman, Aysen Akalın, Erman Çakal, Göknur Yorulmaz, Sema Hepşen, Banu Kara Kıvanç, Neslihan Kurtulmuş, Özlem Zeynep Akyay, Zeliha Hekimsoy, Özlem Turhan İyidir, Fatma Ela Keskin, Zeynel Abidin Sayiner, Ayşe Kubat Üzüm, Ilgın Yıldırım Şimşir, Ceyla Konca Degertekin, Ömercan Topaloğlu, Sevde Nur Fırat, Damla Köksalan, Berrin Çetinarslan, Alev Selek, Emre Gezer, Saadet Acar, Adnan Batman

**Affiliations:** 1 Department of Endocrinology and Metabolism, Kocaeli University Faculty of Medicine, Kocaeli, Türkiye; 2 Department of Endocrinology and Metabolism, Uludag University Faculty of Medicine, Bursa, Türkiye; 3 Department of Endocrinology and Metabolism, Cerrahpasa Faculty of Medicine, Istanbul University Cerrahpasa, Istanbul, Türkiye; 4 Department of Endocrinology and Metabolism, University of Health Sciences, Basaksehir Cam and Sakura City Hospital, Istanbul, Türkiye; 5 Department of Endocrinology and Metabolism, Osmangazi University Faculty of Medicine, Eskişehir, Türkiye; 6 Department of Endocrinology and Metabolism, Ankara Etlik City Hospital, Ankara, Türkiye; 7 Department of Endocrinology and Metabolism, Acibadem Maslak Hospital, Istanbul, Türkiye; 8 Department of Endocrinology and Metabolism, Nev Hospital, Şanlıurfa, Türkiye; 9 Department of Endocrinology and Metabolism, Celal Bayar University Faculty of Medicine, Manisa, Türkiye; 10 Department of Endocrinology and Metabolism, Başkent University Faculty of Medicine, Ankara, Türkiye; 11 Department of Endocrinology and Metabolism, Demiroglu Bilim University Faculty of Medicine, Istanbul, Türkiye; 12 Department of Endocrinology and Metabolism, Gaziantep University Faculty of Medicine, Gaziantep, Türkiye; 13 Department of Endocrinology and Metabolism, Istanbul University Faculty of Medicine, Istanbul, Türkiye; 14 Department of Endocrinology and Metabolism, Ege University Faculty of Medicine, İzmir, Türkiye; 15 Department of Endocrinology and Metabolism, Ufuk University Faculty of Medicine, Ankara, Türkiye; 16 Department of Endocrinology and Metabolism, Zonguldak Bülent Ecevit University Faculty of Medicine, Zonguldak, Türkiye; 17 Department of Endocrinology and Metabolism, Ankara Education and Research Hospital, Ankara, Türkiye; 18 Department of Endocrinology and Metabolism, Derince Education and Research Hospital, Kocaeli, Türkiye; 19 Department of Endocrinology and Metabolism, Acibadem Mehmet Ali Aydinlar University Faculty of Medicine, Istanbul, Türkiye

**Keywords:** bone mineral density, lactation, osteoporosis, osteoporotic fractures, pregnancy, teriparatide

## Abstract

**Background:**

Pregnancy and lactation-associated osteoporosis (PLO) is an uncommon cause of fragility fractures in young women, and the optimal treatment strategy remains unclear. This study aimed to investigate the clinical features, treatment approaches, and bone mineral density (BMD) outcomes of women with PLO across multiple centers in Türkiye.

**Methods:**

This multicenter retrospective cohort study included 59 women with PLO from 18 endocrine centers in Türkiye. Data on demographics, clinical and laboratory results, fractures, treatments, and bone density were collected. Changes in the lumbar spine, femoral neck, and total hip BMD were tracked. Additionally, lumbar spine BMD outcomes were compared between women who received teriparatide and those managed with calcium/vitamin D alone.

**Results:**

The mean age at diagnosis was 29.1 ± 4.8 years. Overall, 86.4% of patients had vertebral fractures, and 67.8% had multiple fractures. Of the 59 patients, 10 received bisphosphonates, 3 received denosumab, 22 received TPTD, and 24 received calcium/vitamin D supplementation alone as first-line treatment. Lumbar spine BMD improved significantly in all treatment groups and total hip BMD in the teriparatide and calcium/vitamin D groups, whereas femoral neck BMD improved significantly only in the teriparatide group. Women receiving TPTD demonstrated greater early improvement in lumbar spine BMD than those managed with calcium/vitamin D alone (19.1% vs 9.5%; Hodges–Lehmann median difference, 11.5 percentage points [95% CI, 1.5 to 22.8]; P = 0.019). However, the between-group difference in lumbar spine BMD improvement was no longer significant at the second follow-up. After adjustment for baseline lumbar spine BMD, age, BMI and multiple vertebral fractures, treatment group was no longer independently associated with early lumbar spine BMD change (B = 3.0 percentage points, 95% CI −12.8 to 18.9; P = 0.698).

**Conclusions:**

Women with PLO showed substantial recovery in bone density during follow-up across treatment groups. Although women receiving TPTD demonstrated greater early lumbar spine BMD improvement in unadjusted analyses, this association was not independent after multivariable adjustment, and between-group differences were no longer observed during later follow-up. Prospective comparative studies are needed to define the optimal treatment strategy.

## 1 Introduction

Pregnancy and lactation-associated osteoporosis (PLO) is a rare condition in young women characterized by low bone mineral density (BMD) and fragility fractures that typically occur during the third trimester of pregnancy or in the early postpartum period (1). Patients usually present with back pain, functional limitation, and height loss due to vertebral fractures, whereas distal radius and sacral fractures are less common (2). Unlike postmenopausal osteoporosis, proximal femur fractures are rare in PLO (3).

The pathophysiology of PLO is incompletely understood. Bone loss during lactation is driven principally by parathyroid hormone-related protein released from the mammary gland and by the fall in estrogen accompanying lactational amenorrhea, which together upregulate receptor activator of nuclear factor-κB ligand and increase osteoclastic resorption (4–6). These changes are physiological and largely reversible after weaning, but may result in fragility fracture in susceptible women. A family history of osteoporosis is the most commonly reported risk factor; previous fracture, smoking, low body mass index, medications and inadequate calcium intake have also been implicated (7, 8).

The optimal management of PLO has not been established. Current treatment includes breastfeeding cessation, calcium and vitamin D supplementation, orthopedic support, and, in selected cases, antiresorptive therapy (bisphosphonates or denosumab) or anabolic therapy with teriparatide (TPTD) (9, 10). Despite the growing awareness of PLO, evidence on its clinical course and optimal management remains limited. Most available data are from case reports, small case series, or single-center studies. Additionally, comparative information on different treatments, such as conservative management, antiresorptive therapy, and TPTD, is limited. Real-world multicenter data needed to better define affected women and evaluate treatment outcomes.

We therefore conducted a nationwide multicenter cohort study in 18 tertiary endocrine centers in Türkiye. We aimed to describe the clinical features and treatment patterns of women with PLO, to characterize longitudinal changes in bone mineral density across three first-line management strategies, and to determine whether treatment strategy is independently associated with early bone density gain after adjustment for baseline bone mineral density and clinical severity.

## 2 Materials and methods

### 2.1 Study design and population

This nationwide multicenter retrospective cohort study included women diagnosed with PLO across 18 tertiary endocrine centers in Türkiye between 2010 and 2022. Medical records were reviewed retrospectively using hospital databases and follow-up files. The primary inclusion criteria were age ≥18 years, occurrence of low-trauma or spontaneous fractures during pregnancy or lactation, and reduced BMD on dual-energy X-ray absorptiometry (DXA). In addition, four women without documented fragility fractures but with markedly reduced bone mass for age (lumbar spine Z-score range −2.9 to −4.7; two women below −4.5), characteristic clinical presentation, and no identifiable secondary cause of osteoporosis were included because they had been clinically diagnosed and managed as having PLO at the participating tertiary endocrine centers. This approach is consistent with proposed diagnostic frameworks for PLO, in which a densitometric threshold in the absence of fracture is accepted when the clinical presentation is characteristic and secondary causes have been excluded (11). The exclusion criteria were treatment with an antiresorptive agent before TPTD treatment, vertebral tumors, history of surgery at the fracture site, bone diseases such as osteomalacia, and missing radiographic data.

### 2.2 Data collection

Data on demographic characteristics, pregnancy-related variables, fracture history, family history, comorbidities, laboratory findings, treatment strategies, and BMD measurements were collected. DXA measurements were obtained from routine clinical practice at each participating center rather than according to a standardized study protocol. The DXA system in use at each participating center is reported in **Supplementary Table S3**. All densitometric measurements for a given woman were obtained at the center at which she was followed. A vertebral fracture was defined as a decrease of ≥20% (+4 mm) in the anterior, middle, or posterior vertebral height on vertebral imaging (12).

BMD results were interpreted using Z-scores according to the International Society for Clinical Densitometry guidelines (13). As follow-up schedules reflected routine clinical practice rather than a study protocol, DXA assessment intervals differed according to the treatment strategy. In Türkiye, the national reimbursement policy routinely covers annual DXA assessments for patients receiving conservative treatment, resulting in follow-up evaluations at approximately 12 and 24 months. In contrast, continuation of teriparatide therapy requires reimbursement-related reassessment with DXA at approximately 6 months, with the subsequent routine follow-up generally performed at approximately 18 months. Therefore, for comparative purposes, these routinely scheduled assessments were categorized as the first and second follow-up visits, respectively. Treatment decisions, including the use of TPTD, antiresorptive agents, and conservative management, were made by the treating physicians in accordance with routine clinical practice. No standardized treatment protocol was applied across the participating centers.

### 2.3 Outcomes

The primary outcomes were clinical characteristics and treatment patterns in women with PLO. The secondary outcomes were longitudinal changes in BMD according to first-line treatment strategy. Comparative analyses were performed between women treated with teriparatide and those receiving calcium/vitamin D alone. In contrast, women receiving antiresorptive therapy were analyzed descriptively because of the small sample size and treatment heterogeneity.

### 2.4 Ethical consideration

This study was approved by the Non-Interventional Clinical Research Ethics Committee of Kocaeli University (Project number 2022/163) and conducted in accordance with the Declaration of Helsinki. The requirement for informed consent was waived due to the retrospective nature of the study.

### 2.5 Statistical analysis

All statistical analyses were performed using IBM SPSS version 29.0 (IBM Corp., Armonk, NY, USA). Data normality was assessed using the Shapiro–Wilk test. Continuous variables were presented as mean ± standard deviation or median and interquartile range (IQR), whereas categorical variables were presented as frequencies and percentages. Comparisons between groups were performed using the independent samples t-test for normally distributed variables and the Mann–Whitney U test for non-normally distributed variables. For non-parametric between-group comparisons, Hodges–Lehmann estimates of the median difference with 95% confidence intervals are reported alongside P values. Associations between categorical variables were assessed using the chi-square test. Comparisons within dependent groups were performed using Friedman’s two-way analysis of variance. Multiple comparisons were performed using Dunn’s test. A P-value < 0.05 indicated statistical significance. Comparisons between treatment groups were considered exploratory due to the observational nature of the study. Longitudinal BMD and Z-score measurements were summarized for all three first-line treatment groups, and overall between-group comparisons were performed using the Kruskal–Wallis test. Because of the small sample size and clinical heterogeneity of the antiresorptive group, adjusted comparative analyses were restricted to women treated with teriparatide and those receiving calcium/vitamin D alone. Propensity score matching was considered but not undertaken, because the available sample size (22 women receiving teriparatide and 24 managed with calcium and vitamin D alone) would have substantially reduced the matched cohort and yielded unstable propensity estimates; multivariable adjustment was therefore preferred. To account for potential confounding due to non-random treatment allocation, a multivariable linear regression analysis was performed with percentage change in lumbar spine BMD from baseline to the first follow-up as the dependent variable. Treatment group (teriparatide vs. calcium/vitamin D), baseline lumbar spine BMD, age, BMI, and multiple vertebral fractures were included as independent variables. As a confirmatory analysis, absolute follow-up BMD was compared between groups using analysis of covariance with baseline BMD, age, BMI and number of prevalent vertebral fractures as covariates. This study is reported in accordance with the STROBE statement for observational studies (**Supplementary Table S2**).

## 3 Results

### 3.1 Baseline characteristics

A total of 59 female patients with a mean age of 29.1 ± 4.8 years were included in this study. The median body mass index was 22.4 kg/m^2^ (IQR, 20.0–25.0 kg/m^2^). Of the 59 patients, 10 (16.9%) had comorbidities and were on regular medication for these conditions. The mean TSH level among patients using levothyroxine was 1.8 ± 0.9 mIU/L (TSH normal reference range: 0.27–4.20 mIU/L). One patient was diagnosed with rheumatoid arthritis and was not receiving active steroid therapy. Only 3 patients had a history of low-molecular-weight heparin (LMWH) use during pregnancy. A total of 41 patients were primiparous, whereas 18 were multiparous. The median breastfeeding duration was 5.0 months (IQR, 3.0–9.0). The most common complaint during admission was back pain (93.2%). The “complaint onset time”, when patients first began to feel their PLO-related complaints, was often in the postpartum period. Furthermore, 4 (6.8%) patients had a history of fractures before pregnancy. A decrease in BMD without any fractures was detected in 4 (6.8%) patients. **Table 1** shows the participants’ baseline clinical characteristics.

**Table 1:** Clinical features of pregnancy and lactation-associated osteoporosis.

Clinical Characteristics	Overall (n = 59)
Age at diagnosis (year)	29.1±4.8
Smoking	6 (10.2%)
Alcohol consumption	3 (5.1%)
Comorbid diseases	10 (16.9%)
Hypothyroidism	4
Rheumatoid arthritis	1
Graves disease (In remission)	1
Migraine	1
PCOS	1
Thalassemia minor	1
Osteogenesis imperfecta (heterozygous mutation)	1
Family history of low-energy fracture	10 (16.9%)
Anticoagulant use in pregnacy	3 (5.1%)
Regular menstrual cycle before pregnancy	53 (89.8%)
Multiparity	18 (30.5%)
Breastfeeding duration (m)	5.0 (3.0–9.0)
Pregnancy type	
Spontaneous	57 (96.6%)
*In vitro* fertilisation	2 (3.4%)
Delivery type	
Vaginal	17 (28.8%)
C/S	42 (71.2%)
Multiple fractures	40 (67.8%)
Fracture location	
Vertebra	51 (86.4%)
Iliac bone	1 (1.7%)
Tarsal bones	2 (3.4%)
Femoral head and distal radius	1 (1.7%)
Vertebral fracture location	
Thoracic	16 (31.4%)
Lumbar	11 (21.6%)
Thoracolumbar	24 (47.1%)
Complaint onset time	
2nd trimester	2 (3.4%)
3rd trimester	23 (39.0%)
Lactation	34 (57.6%)

PCOS, Polycystic ovary syndrome; C/S, Cesarean section.

### 3.2 Laboratory findings

The biochemical analyses conducted at diagnosis revealed mild hypocalcemia (Ca 8.0–8.5 mg/dL, reference range: 8.6–10.6 mg/dL) in only 3 patients and mild hypomagnesemia (Mg 0.8–1.75 mg/dL, reference range: 1.6–2.6 mg/dL) in 7 patients. Among the bone turnover markers, alkaline phosphatase levels were elevated in only 12 patients, with a maximum increase of up to 1.6 times the upper limit of the reference range. The mean serum 25-hydroxyvitamin D concentration at diagnosis was 25.6 ± 11.6 ng/mL (median 24.3, IQR 17.0–31.9). Twenty women (33.9%) had concentrations above 30 ng/mL and 39 (66.1%) had vitamin D insufficiency or deficiency. No other clinically relevant laboratory abnormalities were identified.

### 3.3 Treatment patterns

All patients received calcium and/or vitamin D supplementation as part of routine management. Of the 59 patients, 10 received bisphosphonates, including alendronate (*n* = 7), risedronate (*n* = 2), and zoledronic acid (*n* = 1); 3 received denosumab; 22 received TPTD; and the remaining patients received calcium and vitamin D supplementation only as first-line treatment. Of the 22 patients who received TPTD as first-line treatment, 3 received oral bisphosphonate (alendronate), and 1 received denosumab as second-line treatment. The patient who was initially treated with zoledronic acid received TPTD as second-line treatment. The mean duration of TPTD treatment was 13.4 ± 5.8 months (range, 4–18 months). Of the 22 women who received TPTD, 15 (68.2%) received treatment for at least 12 months, and 11 (50.0%) completed the 18-month course reimbursed under national coverage criteria in Türkiye. In the remaining 11 women (50.0%), treatment was discontinued earlier than intended. The most common reason for early discontinuation was reimbursement restrictions (n = 7), followed by patient preference (n = 2) and loss to follow-up (n = 2). No patient discontinued treatment because of an adverse drug reaction. Of the 5 patients who received second-line treatment, treatment was changed in 2 due to new vertebral fractures and in 3 due to a lack of significant bone mineral gain.

### 3.4 BMD outcomes

A first follow-up densitometric measurement was available for 52 of 59 women and a second for 32 (calcium/vitamin D 11 of 24, antiresorptive 6 of 13, teriparatide 15 of 22; **Figure 1**; **Supplementary Table S1**). Because attrition was greatest at the second visit, the adjusted comparison at the first follow-up, in which 39 women contributed paired lumbar spine measurements, provides the primary basis for inference. BMD was assessed using DXA to evaluate treatment responses (**Figure 2**). Measurement results were obtained for up to 2 years. A total of 13 patients received antiresorptive therapy using various agents. **Table 2** presents the longitudinal BMD and Z-score measurements according to the three first-line treatment strategies (calcium/vitamin D alone, antiresorptive therapy, and teriparatide). Baseline lumbar spine BMD and Z-scores differed significantly across the three groups, with the lowest values observed in women treated with teriparatide. No statistically significant overall between-group differences were observed at the first or second follow-up visits. Compared with baseline, lumbar spine BMD increased significantly over time in all three treatment groups (**Table 2**). **Table 3** shows the percentage changes in BMD from baseline to the first and second follow-up visits according to first-line treatment strategy. Evaluation of percentage changes revealed that improvements in lumbar spine BMD at the first follow-up were significantly greater in those receiving TPTD than in those receiving calcium and vitamin D only (**Figure 3**). The percentage improvement at the second follow-up continued to numerically favor TPTD; however, the difference was not statistically significant (**Table 3**, **Figure 2**). This finding is consistent with the lower baseline lumbar spine BMD observed in the teriparatide group, reflecting the tendency for anabolic therapy to be prescribed to women with more severe skeletal involvement.

**Figure 1:**
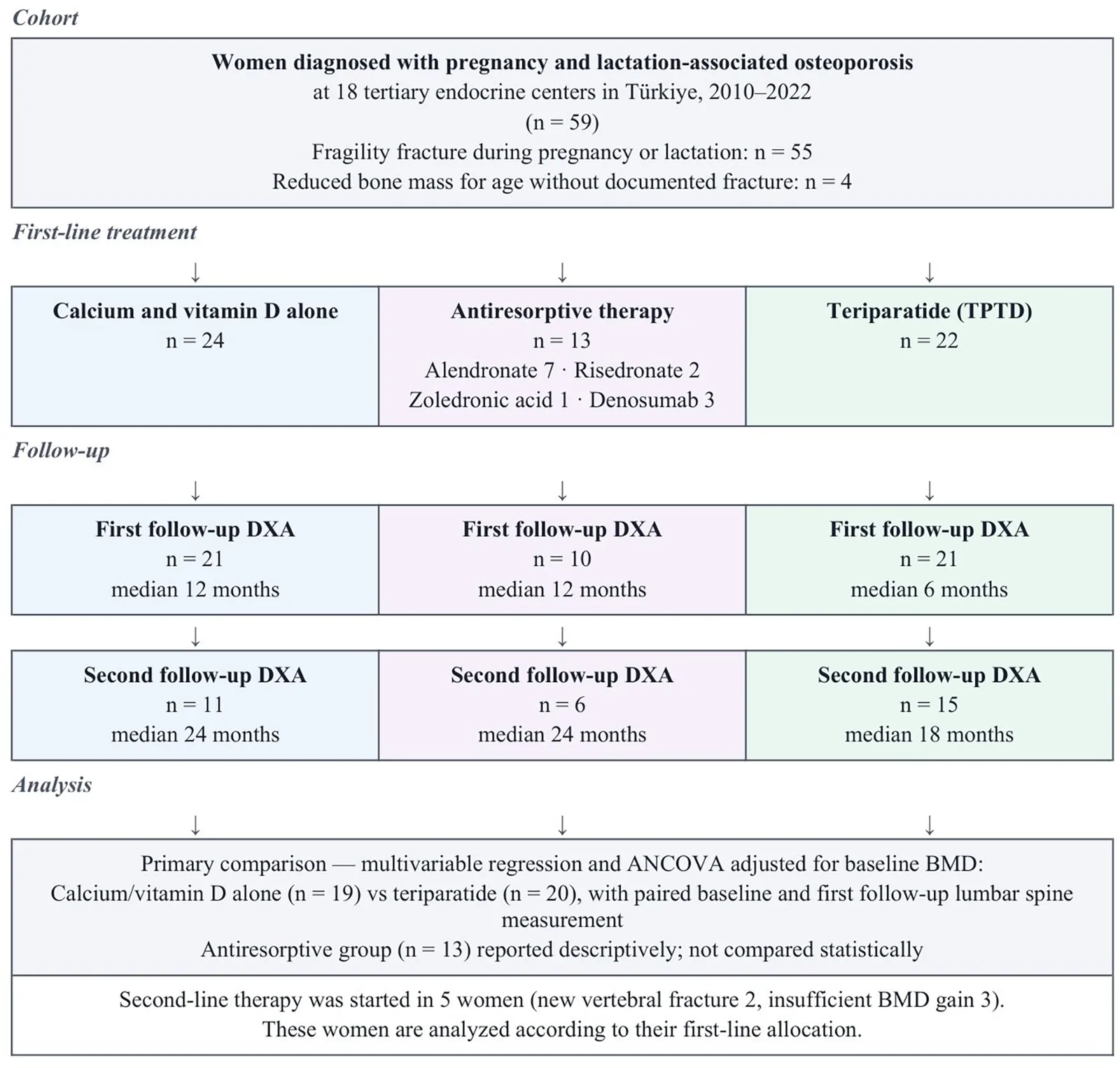
Flow of participants through the study. Allocation of the 59 women with pregnancy and lactation-associated osteoporosis to the three first-line treatment strategies, and the number contributing an interpretable lumbar spine densitometric measurement at each follow-up visit. Median intervals are calculated from the baseline scan. Denominators for the femoral neck and total hip are given in **Supplementary Table S1**. Because the cohort was assembled retrospectively from women already diagnosed and managed as having PLO at the participating centers, the number of records screened before inclusion could not be reconstructed. Second-line therapy was started in five women, who are analyzed according to their first-line allocation. PLO, pregnancy and lactation-associated osteoporosis; DXA, dual-energy X-ray absorptiometry; TPTD, teriparatide.

**Figure 2:**
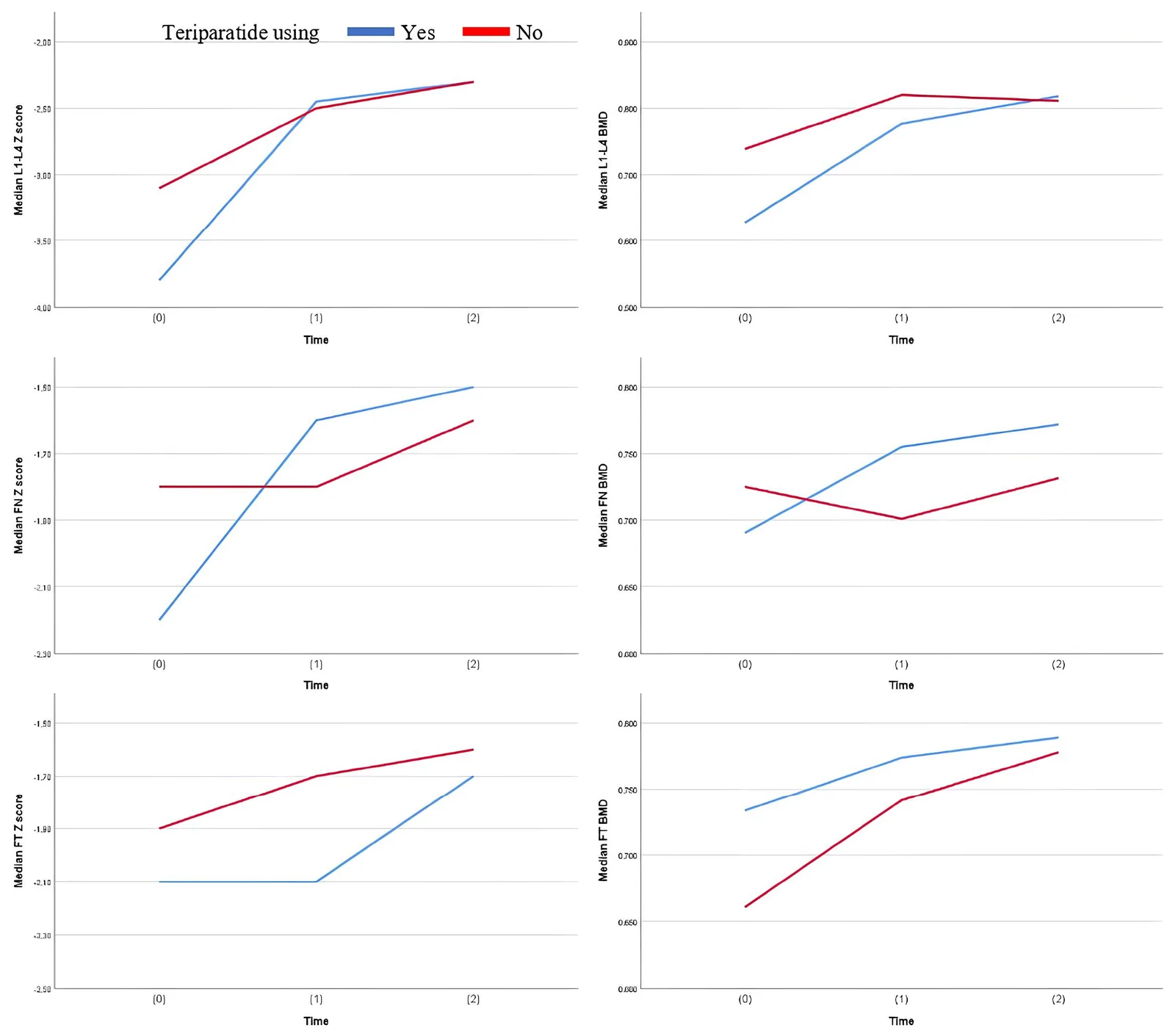
Longitudinal changes in median bone mineral density (BMD) and Z-scores according to teriparatide treatment. Median lumbar spine (L1–L4), femoral neck (FN), and total hip (TH) BMD and Z-scores at baseline (0), first follow-up (1), and second follow-up (2) in women who received teriparatide (blue) and those managed without teriparatide (red). BMD values are expressed in g/cm^2^.

**Table 2:** Bone mineral density and Z-scores at baseline and follow-up, by treatment group.

Site	Measure	Visit	Calcium/vitamin D (n = 24)	P †	Antiresorptive (n = 13)	P †	Teriparatide (n = 22)	P †	P *
Lumbar spine (L1–L4)	BMD (g/cm^2^)	0	0.765 (0.718–0.847), 19	0.001	0.705 (0.653–0.732), 10	0.006	0.628 (0.577–0.720), 20	< 0.001	0.001
		1	0.833 (0.772–0.872), 21		0.798 (0.781–0.847), 10		0.777 (0.705–0.835), 21		0.172
		2	0.828 (0.786–0.942), 11		0.810 (0.750–0.818), 6		0.818 (0.766–0.845), 15		0.484
Lumbar spine (L1–L4)	Z-score	0	−3.00 (−3.52 to −2.58), 24	< 0.001	−3.40 (−3.80 to −3.10), 13	0.007	−3.80 (−4.47 to −3.28), 22	< 0.001	0.002
		1	−2.40 (−2.90 to −1.88), 24		−2.60 (−3.10 to −2.00), 13		−2.45 (−3.48 to −2.21), 22		0.261
		2	−2.25 (−2.55 to −1.45), 12		−2.90 (−3.20 to −2.10), 9		−2.30 (−2.70 to −1.71), 15		0.204
Femoral neck	BMD (g/cm^2^)	0	0.725 (0.621–0.749), 19	0.202	0.732 (0.697–0.772), 10	0.311	0.691 (0.653–0.760), 20	0.032	0.550
		1	0.669 (0.616–0.814), 19		0.759 (0.701–0.810), 10		0.755 (0.698–0.802), 21		0.330
		2	0.722 (0.682–0.744), 10		0.744 (0.718–0.787), 6		0.772 (0.702–0.826), 15		0.264
Femoral neck	Z-score	0	−1.80 (−2.35 to −1.25), 24	0.033	−1.90 (−2.80 to −1.10), 13	0.236	−2.20 (−2.58 to −1.52), 22	0.003	0.798
		1	−1.95 (−2.27 to −1.12), 22		−1.70 (−1.90 to −1.10), 13		−1.60 (−2.35 to −1.12), 22		0.807
		2	−1.90 (−2.10 to −1.15), 11		−1.40 (−2.10 to −1.10), 9		−1.50 (−2.00 to −1.00), 15		0.767
Total hip	BMD (g/cm^2^)	0	0.686 (0.624–0.766), 19	0.002	0.638 (0.581–0.769), 10	0.115	0.734 (0.595–0.788), 20	0.005	0.672
		1	0.739 (0.695–0.821), 20		0.755 (0.692–0.808), 10		0.774 (0.672–0.802), 21		0.990
		2	0.796 (0.732–0.817), 11		0.744 (0.704–0.813), 6		0.789 (0.736–0.803), 15		0.730
Total hip	Z-score	0	−1.90 (−2.42 to −0.97), 20	0.006	−1.90 (−2.80 to −1.18), 10	0.070	−2.10 (−3.02 to −1.70), 20	0.007	0.152
		1	−1.70 (−2.30 to −1.20), 21		−1.75 (−2.15 to −1.25), 10		−2.10 (−2.60 to −1.60), 21		0.232
		2	−1.60 (−2.05 to −0.93), 12		−1.55 (−2.02 to −1.15), 6		−1.70 (−2.10 to −1.40), 15		0.615

Values are median (interquartile range), followed by the number of women contributing data. Denominators differ between cells because not all women had an interpretable measurement at every visit; see **Supplementary Table S1**.

*Between-group comparison at each visit (Kruskal–Wallis test). † Within-group comparison across the three visits (Friedman test), restricted to women with measurements at all three time points; the P value is shown once per measure and applies to the three visits jointly.

The number of women contributing data differs across visits because not all participants underwent DXA assessment at every follow-up visit (see **Supplementary Table S1**).

Visits: 0 Baseline; 1 First follow-up; 2 Second follow-up.

**Table 3:** Percentage changes in bone mineral density from baseline by first-line treatment group.

Site	Interval	Calcium/vitamin D percentage change	Antiresorptive rcentage change	Teriparatide percentage change	Unadjusted difference, TPTD vs Ca/vitD (95% CI) †	P
Lumbar spine (L1–L4)	Visit 1	9.5 (2.3–18.1), 19	12.8 (5.5–24.7), 10	19.1 (9.3–33.4), 20	+11.5 (+1.5 to +22.8)	0.019
Lumbar spine (L1–L4)	Visit 2	15.4 (6.0–23.0), 10	7.2 (2.5–26.0), 6	31.1 (11.1–47.0), 14	+13.0 (-0.9 to +32.1)	0.107
Femoral neck	Visit 1	4.3 (-2.1–8.4), 19	5.7 (-0.9–14.8), 10	3.9 (1.4–9.7), 20	+0.2 (-4.8 to +8.3)	0.922
Femoral neck	Visit 2	10.1 (-1.0–12.2), 10	4.1 (1.6–6.9), 6	5.2 (-0.1–17.3), 14	+1.3 (-10.4 to +9.3)	0.930
Total hip	Visit 1	5.7 (4.1–15.6), 19	8.7 (4.6–20.4), 10	3.2 (0.1–7.3), 20	-4.5 (-10.8 to +0.2)	0.066
Total hip	Visit 2	13.7 (4.7–34.6), 10	9.9 (5.5–18.9), 6	4.8 (1.8–9.8), 14	-8.7 (-28.4 to +3.0)	0.151

Values are presented as median (interquartile range), with the number of women contributing paired measurements shown for each cell. The antiresorptive group is presented descriptively and was not included in formal comparative analyses. Between-group comparisons (TPTD vs calcium/vitamin D) were performed using the Mann–Whitney U test. Hodges–Lehmann median differences with 95% confidence intervals are reported.

**Figure 3:**
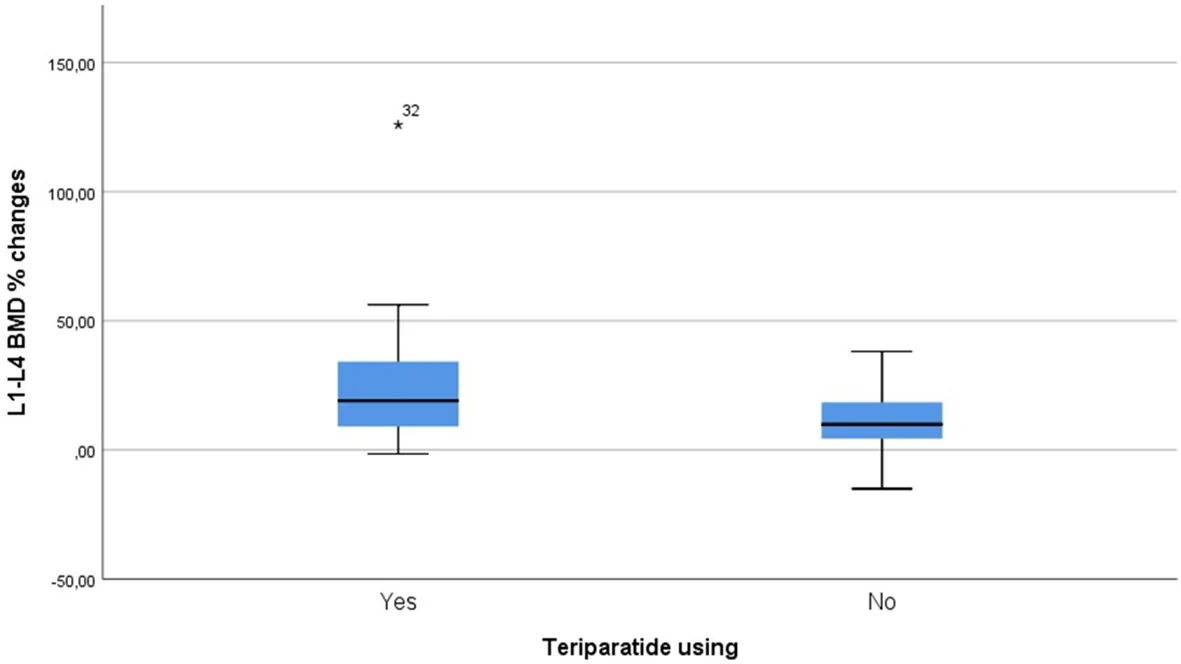
Percentage change in lumbar spine (L1–L4) bone mineral density (BMD) according to teriparatide treatment. Box-and-whisker plots show the percentage change in lumbar spine BMD from baseline to the first follow-up in patients who received teriparatide and those who did not. The center line represents the median, the box indicates the interquartile range (IQR), whiskers represent the minimum and maximum values excluding outliers, and the asterisk indicates an outlier. Women treated with teriparatide demonstrated significantly greater percentage improvement in lumbar spine BMD at the first follow-up than women managed without teriparatide. This difference was not independent of baseline lumbar spine BMD in multivariable analysis (**Table 4**).

Total hip BMD improved significantly over time in both the teriparatide and calcium/vitamin D groups, whereas femoral neck BMD improved significantly only in the teriparatide group. However, no significant between-group differences in percentage changes were observed at either follow-up (**Table 3**, **Figure 2**). The antiresorptive group is presented descriptively in **Tables 2**, **3**. Because of the small sample size and pharmacological heterogeneity of antiresorptive therapy, this group was not included in the formal comparative analyses.

To address potential confounding due to baseline differences between the teriparatide and calcium/vitamin D groups, a multivariable linear regression analysis was performed (**Table 4**). After adjustment for baseline lumbar spine BMD, age, BMI, and multiple vertebral fractures, treatment group was not independently associated with percentage lumbar spine BMD change at the first follow-up (β = 0.066, B = 3.045, 95% CI −12.763 to 18.853, p = 0.698). Baseline lumbar spine BMD remained the only independent predictor of percentage lumbar spine BMD change (β = −0.595, B = −108.504, 95% CI −169.013 to −47.994, p = 0.001). The overall regression model was statistically significant (F = 5.358, p = 0.001; adjusted R^2^ = 0.364). Because percentage change is mathematically dependent on the baseline value, the analysis was repeated as an analysis of covariance modelling absolute follow-up BMD with baseline BMD as a covariate. This yielded the same conclusion: the adjusted between-group difference in lumbar spine BMD was +0.011 g/cm^2^ (95% CI −0.081 to +0.103; P = 0.810) at the first follow-up and −0.013 g/cm^2^ (95% CI −0.080 to +0.055; P = 0.700) at the second.

**Table 4:** Multivariable linear regression analysis for percentage change in lumbar spine BMD from baseline to first follow-up.

Variable	B (95% CI)	Standardized β	p value
Treatment (TPTD vs calcium/vitamin D)	3.045 (−12.763 to 18.853)	0.066	0.698
Baseline lumbar spine BMD	−108.504 (−169.013 to −47.994)	−0.595	0.001
Age	−0.214 (−1.595 to 1.167)	−0.042	0.754
BMI	−0.521 (−2.266 to 1.223)	−0.086	0.548
Multiple vertebral fractures	−12.185 (−26.244 to 1.875)	−0.236	0.087

Overall model: F = 5.358, p = 0.001; R^2^ = 0.448; Adjusted R^2^ = 0.364. The multivariable regression analysis included 39 women with complete data for all variables entered into the model.

## 4 Discussion

This nationwide study of women with PLO showed that vertebral fractures were the most prevalent clinical manifestation. Additionally, many women had low vitamin D levels at the time of diagnosis. The high number of patients with vertebral and multiple fractures indicates that PLO can seriously affect bone health, even in younger women. BMD increased during follow-up across the treatment groups. In unadjusted analyses, women receiving TPTD showed a greater increase in lumbar spine BMD at the first follow-up; however, treatment group was not independently associated with this change after multivariable adjustment. These findings suggest that substantial bone density recovery is possible in women with PLO. The apparent early advantage of TPTD should be interpreted cautiously because treatment allocation was non-random and patients receiving TPTD had more severe skeletal involvement at baseline.

Physiological changes during pregnancy and lactation increase maternal calcium demand and can result in temporary skeletal loss, particularly during lactation (14). Low pre-pregnancy BMD, mechanical pressure on the spine, insufficient calcium intake and absorption, genetic factors, vitamin D deficiency, hypercalciuria, and pharmacotherapy (e.g., LMWH, steroids, and anticonvulsants) have been reported to contribute to the pathogenesis of PLO (1). Increased parathyroid hormone-related protein secretion from the mammary gland, hypoestrogenism related to lactational amenorrhea, and enhanced osteoclast activity through receptor activator of nuclear factor-κB ligand-mediated pathways have been implicated in PLO (4, 5). Breastfeeding stimulates the release of pituitary prolactin, and increased prolactin inhibits the secretion of gonadotropin-releasing hormone. Inhibition of gonadotropin-releasing hormone secretion reduces ovarian sex steroid levels, including estradiol and progesterone (6). The decrease in plasma estrogen levels and the increase in parathyroid hormone-related protein levels secreted by the mammary gland lead to upregulation of receptor activator of nuclear factor-κB ligand, thereby stimulating osteoclast development and bone resorption (1, 6). These changes are reversible in most women after weaning. However, they may contribute to clinically significant bone loss and fragility fractures in susceptible individuals.

Heparin is another factor that contributes to the pathogenesis of PLO. Heparin increases bone resorption by directly affecting bone cells, reducing osteoblastic activity, and increasing osteoclastic activity (15). LMWH is less likely to cause osteoporosis than standard heparin (16). A recent systematic review identified the use of heparin or LMWH in 17 patients with PLO. However, the number of patients who used heparin versus LMWH was not specified (17). A prospective observational study showed that bone loss due to long-term LMWH use at a dose of 5000 IU/day throughout pregnancy did not differ significantly from the physiological losses observed during pregnancy (18). In our study, only three patients had a history of LMWH use.

The most common complaints of women with PLO during admission are back pain and loss of height due to vertebral fractures (14). Fractures in these patients occur more frequently in the vertebrae than in other sites (19). A previous study evaluating the bone microarchitecture of women with PLO using high-resolution peripheral quantitative computed tomography showed that the average volumetric BMD of the distal radius was 25.0% lower than that in the control group. Additionally, the trabecular compartment was more affected than the cortical compartment in these patients (20). In the same study, areal BMD assessed by DXA was 32.0% lower in the lumbar spine and 24.7% lower in the femoral neck in women with PLO. Similarly, another study evaluating vertebral bone density using QCT showed that lumbar vertebral areal and volumetric BMD were significantly lower in women with PLO (21). The greater bone loss observed in the vertebrae than in other sites in women with PLO is consistent with vertebral fractures. Consistent with the literature, our results showed that the vertebrae were the most common fracture site. Furthermore, the predominance of thoracolumbar involvement in our cohort is clinically relevant, as this region is particularly vulnerable to mechanical loading during late pregnancy and the early postpartum period.

In this study, most women exhibited low or insufficient vitamin D levels, consistent with previous findings indicating that inadequate vitamin D impairs bone adaptation during pregnancy and lactation. Vitamin D deficiency alone is unlikely to cause PLO. However, insufficient vitamin D may exacerbate bone loss in at-risk women by disrupting calcium homeostasis and impairing bone recovery. These findings indicate that assessment and correction of vitamin D deficiency should be routinely incorporated into the evaluation and management of women with PLO (7, 22). This high prevalence may also reflect regional and lifestyle-related factors. Although Türkiye is located in the Mediterranean region, cultural factors may limit sun exposure. Additionally, the dietary habits of pregnant women and reduced physical activity due to greater weight gain during the second or third pregnancy may contribute to the observed differences.

The lack of a standardized treatment for PLO is the greatest challenge in managing these patients. Although back pain associated with vertebral fractures can be managed with analgesics, physical therapy, bed rest, and vertebroplasty, no consensus has been reached on the treatment indications for low BMD, the optimal type of therapy, and its duration. Some clinicians adopt an observational approach along with breastfeeding cessation and delay specific treatment. A previous study showed an annual increase of 6.6% in vertebral BMD and 2.3% in femoral neck BMD in patients who received no specific therapy during a 2.5-year follow-up (14). Orhadje et al. reported that patients receiving calcium and vitamin D supplementation achieved a 10.8 ± 8.5% increase in vertebral BMD, a 4.9 ± 3.3% increase in femoral neck BMD, and a 6.8 ± 2.8% increase in total femoral BMD compared with baseline (23). A recent meta-analysis showed that calcium monotherapy resulted in increases of 2.0% and 4.9%–11.0% in vertebral BMD at 12 and 24 months, respectively, and an increase of 14.4% in femoral neck BMD at 24 months. Furthermore, the combination of calcium and vitamin D increased vertebral BMD by 2.0%–7.5% at 12 months, 11.0%–12.2% at 24 months, and 41.8% at 36 months and increased femoral neck BMD by 6.1% at 18 months (24). Our results showed significant improvements over time in lumbar spine and total hip BMD in women receiving calcium and vitamin D supplementation. The predominance of lumbar spine BMD improvement observed in our cohort is consistent with previous HR-pQCT studies suggesting greater trabecular than cortical skeletal involvement in women with PLO (20). These findings support the concept that spontaneous skeletal recovery after lactation cessation, together with calcium and vitamin D supplementation, may contribute to BMD improvement in women with PLO.

The main treatment for bone healing in women with PLO is to provide calcium and vitamin D supplements and stop breastfeeding. Some experts report that the use of antiresorptive or anabolic therapy helps recovery and lowers the chance of future fractures (22, 25, 26). TPTD is a recombinant human parathyroid hormone with anabolic effects on bone and is one of the preferred treatment agents for patients with PLO. Histomorphometric studies have shown that intermittent rhPTH therapy rapidly increases bone formation markers and stimulates bone formation before resorption. This interval in which the anabolic effect of rhPTH is dominant can be defined as an “anabolic window.” In this period, rhPTH exerts its maximum anabolic effect (27). A previous case series showed that 18 months of TPTD therapy increased vertebral BMD by 14.5%–25.0% and femoral neck BMD by 9.5%–16.7% (28). These findings are supported by a recent meta-analysis, which reported that TPTD therapy improved vertebral and femoral neck BMD by up to 36.0% and 18.6%, respectively. However, the authors emphasized that no firm conclusions can be drawn regarding the optimal therapeutic intervention in women with PLO due to substantial heterogeneity and the lack of robust comparative data (24). Furthermore, Adamidou et al. reported that patients receiving TPTD in addition to calcium and vitamin D treatment demonstrated a mean increase in vertebral BMD of 20.9 ± 11.9% at 12 months, whereas those not receiving TPTD showed only a mean increase of 6.2 ± 4.8%, with a statistically significant difference. However, no significant improvement in the total femoral BMD was observed (25). Hong et al. reported significantly higher increases in vertebral and femoral BMD in patients who received TPTD for 1 year than in those who received conventional therapy (29). It is worth noting that these earlier comparisons, including those of Adamidou et al. (25) and Hong et al. (29), were based on percentage changes from baseline, and the treatment groups were neither balanced nor statistically adjusted for baseline bone density. Our findings raise the possibility that the reported differences may partly reflect the mathematical dependence of percentage change on the baseline value rather than an independent anabolic treatment effect, and suggest that baseline-adjusted comparisons are needed before the superiority of anabolic therapy in PLO can be regarded as established. In our cohort, unadjusted analyses showed greater early improvement in lumbar spine BMD among women treated with TPTD. However, after adjustment for baseline lumbar spine BMD, age, BMI, and multiple vertebral fractures, treatment was no longer independently associated with early lumbar spine BMD improvement. These findings suggest that the apparent early advantage of TPTD may largely reflect the lower baseline lumbar spine BMD and greater disease severity of patients selected for anabolic therapy in routine clinical practice rather than an independent treatment effect. During up to two years of follow-up, BMD values in the two groups converged. This convergence is more consistent with the natural recovery of PLO after weaning occurring in both groups, together with the finite duration of anabolic therapy, than with the loss of an independent treatment effect, given that the apparent early advantage of TPTD was no longer independent of baseline lumbar spine BMD after adjustment.

The choice of outcome metric proved decisive in our cohort. Expressed as percentage change from baseline, the early gain in lumbar spine BMD favored TPTD and was statistically significant; expressed as absolute change in g/cm^2^, the same comparison was no longer significant; and after adjustment for baseline BMD the difference disappeared entirely. This pattern is consistent with the combined effects of baseline imbalance, the mathematical dependence of percentage change on the baseline value, and regression to the mean. Because percentage change is obtained by dividing by the baseline value, a given absolute gain is arithmetically inflated when the starting density is low, and women receiving TPTD began from a median lumbar spine BMD of 0.628 g/cm^2^ compared with 0.765 g/cm^2^ in those managed with calcium and vitamin D alone. Percentage change therefore remains useful for comparability with earlier reports, but it is not a sound basis for inferring treatment effect when the groups differ substantially at baseline.

Women receiving antiresorptive therapy also demonstrated improvements in lumbar spine and hip BMD during follow-up. However, because this group comprised a small number of women treated with pharmacologically heterogeneous agents, these findings are presented descriptively and should not be interpreted as evidence of comparative treatment efficacy. Nevertheless, they provide additional real-world information regarding treatment patterns in women who did not receive teriparatide.

Unlike postmenopausal osteoporosis, patients with PLO, including those with fractures, are likely to experience spontaneous recovery and maintain their BMD after the cessation of childbirth and lactation, as factors contributing to the pathogenesis are eliminated and adequate estrogen levels are restored (1, 5). This biological reversibility may partly explain why substantial BMD recovery was observed in all three treatment groups, including women who received calcium and vitamin D alone. The major limitation of TPTD therapy is that the approved treatment duration is a maximum of 24 months (30). Several studies have shown that the most potent effects of TPTD on bone formation markers occur within the first 6–12 months and then begin to decline (31, 32). Therefore, three main questions remain regarding TPTD treatment in patients with PLO: How long should the treatment last? Is sequential therapy required? If a new pregnancy is planned, how long should treatment be discontinued before the subsequent pregnancy? (33). Given the lack of clear treatment guidelines for using TPTD in women with PLO, our findings indicate that treatment should be tailored to each patient, and decisions should consider fracture burden, baseline BMD, symptom severity, future pregnancy plans, and the patient’s overall clinical response.

This study has some limitations. First, analyses of potential risk factors were limited by the retrospective multicenter design. Second, treatment allocation was not randomized, as physicians made decisions based on their routine clinical practice, which may have introduced selection bias. Notably, the TPTD group had a lower baseline lumbar spine BMD, suggesting more severe skeletal involvement at diagnosis. Third, DXA measurements were performed at several centers using scanners from different manufacturers as part of routine clinical practice (**Supplementary Table S3**). Model designations, software versions, local quality assurance procedures and calibration records were not available because of the retrospective design, and cross-calibration between scanners was not feasible. Because each woman was scanned at the center at which she was followed, within-patient change—the basis of all comparisons reported here—is expected to be less susceptible to inter-device differences than pooled absolute BMD values across centers. Nevertheless, absolute BMD values and Z-scores pooled across centers should be interpreted with the possibility of systematic inter-device differences in mind. In addition, follow-up DXA assessments were performed according to routine reimbursement-based clinical schedules rather than a standardized research protocol. Therefore, follow-up intervals differed between treatment groups and should be considered when interpreting comparative analyses. Fourth, only a small number of patients received heterogeneous antiresorptive therapies, limiting comparative analyses. Fifth, the duration of vitamin D, calcium, and TPTD treatment varied because patients were managed by different physicians. Sixth, data on subsequent pregnancies and pregnancy outcomes were not systematically collected because of the retrospective multicenter design. Finally, because of the retrospective multicenter design, data on incident fractures, pain, functional status, quality of life, and bone turnover markers were not systematically or consistently collected during follow-up, precluding comparisons of these clinically important outcomes between treatment groups. In addition, the relatively small sample size may have limited the statistical power to detect modest between-group differences. For the group sizes contributing paired lumbar spine measurements (19 vs 20) and the observed variability (SD 23.5%), only a standardized difference of 0.90 — approximately 21 percentage points of BMD change — would have been detectable with 80% power. Therefore, non-significant findings should not be interpreted as evidence of equivalence between treatment strategies, and the possibility of a type II error should be considered when interpreting these results. Despite these limitations, the strengths of this study lie in its inclusion of a nationwide multicenter cohort of women with a rare skeletal disorder and its provision of real-world data on clinical presentation, treatment patterns, and changes in BMD over time with different management approaches.

## 5 Conclusion

Although women treated with TPTD demonstrated greater early lumbar spine BMD improvement in the unadjusted analysis, this association was not independent after adjustment for baseline lumbar spine BMD and other clinical covariates. During up to two years of follow-up, BMD values in the treatment groups converged, and no independent effect of anabolic therapy on bone density trajectory could be demonstrated. These findings support an individualized treatment approach based on disease severity, fracture burden, baseline BMD, clinical response, and future pregnancy plans. Prospective comparative studies with fracture outcomes are needed before one management strategy can be considered superior to another.

## Data Availability

The data analyzed in this study is subject to the following licenses/restrictions: The dataset contains de-identified patient data but is not publicly available due to ethical and privacy restrictions. Data may be made available from the corresponding author upon reasonable request and with approval from the relevant ethics committee. Requests to access these datasets should be directed to Mehmet Sözen, mehmetsozen07@gmail.com.
