# Supplementary material for: Real-world treatment outcomes in pregnancy and lactation-associated osteoporosis: a nationwide multicenter cohort study
Source: Front Endocrinol (Lausanne). 2026 Sep 17;17:1934166. doi: 10.3389/fendo.2026.1934166 (PMC13626871; doi:10.3389/fendo.2026.1934166)
Supplement: Supplementary file 1 [file Table1.docx]

Supplementary Material

**Supplementary Table S1.** Availability of DXA measurements during follow-up

| Treatment group | Women | Lumbar spine baseline / visit 1 / visit 2 | Femoral neck baseline / visit 1 / visit 2 | Total hip baseline / visit 1 / visit 2 | No second DXA |
| --- | --- | --- | --- | --- | --- |
| Calcium/vitamin D alone | 24 | 19 / 21 / 11 | 19 / 19 / 10 | 19 / 20 / 11 | 13 |
| Antiresorptive therapy | 13 | 10 / 10 / 6 | 10 / 10 / 6 | 10 / 10 / 6 | 7 |
| Teriparatide | 22 | 20 / 21 / 15 | 20 / 21 / 15 | 20 / 21 / 15 | 7 |

Women without a second densitometric measurement did not differ significantly from those with complete follow-up in age (P = 0.88), body mass index (P = 0.82), baseline lumbar spine BMD (P = 0.38), number of prevalent fractures (P = 0.59) or breastfeeding duration (P = 0.93; Mann–Whitney U test), consistent with data missing at random. Some women had an interpretable follow-up measurement without an interpretable baseline scan at the same site; denominators therefore vary between visits.

**Supplementary Table S2. STROBE statement — checklist of items that should be included in reports of cohort studies**

| Section | Item | Recommendation | Reported in | Page / line |
| --- | --- | --- | --- | --- |
| Title and abstract | 1a | Indicate the study's design with a commonly used term in the title or the abstract | Title: “A Nationwide Multicenter Cohort Study”. Abstract, Methods: “multicenter retrospective cohort study”. |  |
|  | 1b | Provide in the abstract an informative and balanced summary of what was done and what was found | Abstract. Both the unadjusted between-group difference and the null result after multivariable adjustment are reported. |  |
| Introduction | 2 | Explain the scientific background and rationale for the investigation being reported | Introduction, paragraphs 1–3. |  |
|  | 3 | State specific objectives, including any prespecified hypotheses | Introduction, final paragraph. Section 2.3 states that comparative analyses were exploratory; no hypothesis was prespecified. |  |
| Methods | 4 | Present key elements of study design early in the paper | Section 2.1, first sentence. |  |
|  | 5 | Describe the setting, locations, and relevant dates, including periods of recruitment, exposure, follow-up, and data collection | Section 2.1: 18 tertiary endocrine centers in Türkiye, 2010–2022. Section 2.2 describes follow-up scheduling. |  |
|  | 6a | Give the eligibility criteria, and the sources and methods of selection of participants; describe methods of follow-up | Section 2.1. Inclusion and exclusion criteria, including the four women without documented fracture. Follow-up described in Section 2.2. |  |
|  | 6b | For matched studies, give matching criteria and number of exposed and unexposed | Not applicable; no matching was performed. Section 2.5 explains why propensity score matching was not undertaken. |  |
|  | 7 | Clearly define all outcomes, exposures, predictors, potential confounders, and effect modifiers | Section 2.2 (vertebral fracture definition, BMD assessment), Section 2.3 (outcomes), Section 2.5 (covariates entered into the adjusted models). |  |
|  | 8 | For each variable of interest, give sources of data and details of methods of assessment; describe comparability of assessment methods if there is more than one group | Section 2.2. DXA performed at each participating center as part of routine care; the system in use at each center is reported. Z-scores interpreted according to ISCD guidance. Model designations, software versions and calibration records were not retrievable, and no cross-calibration was performed; this is stated in the Limitations. |  |
|  | 9 | Describe any efforts to address potential sources of bias | Section 2.5 (multivariable adjustment, confirmatory ANCOVA, rationale against propensity score matching). Discussion and Limitations address confounding by indication and differential follow-up intervals. |  |
|  | 10 | Explain how the study size was arrived at | Section 2.1 and Limitations. All eligible patients identified at the participating centers were included; no a priori sample size calculation was performed. The minimum detectable difference for the achieved sample is reported in the Limitations. |  |
|  | 11 | Explain how quantitative variables were handled in the analyses; describe groupings and why | Section 2.5. BMD analyzed both as percentage change (descriptive) and as absolute value with baseline as a covariate (inferential); rationale given in the Discussion. |  |
|  | 12a | Describe all statistical methods, including those used to control for confounding | Section 2.5. |  |
|  | 12b | Describe any methods used to examine subgroups and interactions | Section 2.5. Three first-line treatment groups are described; the antiresorptive group is reported descriptively and was not compared statistically. |  |
|  | 12c | Explain how missing data were addressed | Section 3.4 reports the number of women contributing data at each visit. Denominators are given in Table 2 and Supplementary Table S1. Missing values were not imputed. |  |
|  | 12d | If applicable, explain how loss to follow-up was addressed | Section 3.4 and Supplementary Table S1. Women without a second densitometric measurement did not differ from those with complete follow-up in age, body mass index, baseline lumbar spine BMD, number of prevalent fractures or breastfeeding duration. |  |
|  | 12e | Describe any sensitivity analyses | Section 2.5 and Section 3.4. The adjusted comparison was repeated as an analysis of covariance on absolute follow-up BMD. |  |
| Results | 13a | Report numbers of individuals at each stage of the study | Figure 1 and Supplementary Table S1. Numbers included, allocated to each first-line strategy, and contributing measurements at each visit. |  |
|  | 13b | Give reasons for non-participation at each stage | Figure 1 legend. The cohort was assembled retrospectively from patients already diagnosed and managed as having PLO; the number of records screened before inclusion could not be reconstructed. |  |
|  | 13c | Consider use of a flow diagram | Figure 1. |  |
|  | 14a | Give characteristics of study participants and information on exposures and potential confounders | Section 3.1 and Table 1. |  |
|  | 14b | Indicate number of participants with missing data for each variable of interest | Table 2 and Supplementary Table S1 give the number of women contributing each densitometric measurement at each visit. |  |
|  | 14c | Summarise follow-up time | Section 2.2 and Figure 1. Median intervals from baseline are reported for each treatment group at each follow-up visit. |  |
|  | 15 | Report numbers of outcome events or summary measures over time | Section 3.4, Table 2 and Table 3. Incident fractures during follow-up were not systematically recorded; this is stated in the Limitations. |  |
|  | 16a | Give unadjusted estimates and, if applicable, confounder-adjusted estimates and their precision; make clear which confounders were adjusted for and why they were included | Section 3.4, Table 3 and Table 4. Unadjusted differences are reported with Hodges–Lehmann estimates and 95% confidence intervals; adjusted estimates are reported with 95% confidence intervals and the covariate set is specified. |  |
|  | 16b | Report category boundaries when continuous variables were categorized | Section 3.2 (vitamin D thresholds) and Section 2.2 (Z-score interpretation). |  |
|  | 16c | If relevant, consider translating estimates of relative risk into absolute risk for a meaningful time period | Not applicable; no relative risk estimates were derived. |  |
|  | 17 | Report other analyses done | Section 3.4. Confirmatory analysis of covariance and descriptive comparison across the three first-line treatment groups. |  |
| Discussion | 18 | Summarise key results with reference to study objectives | Discussion, first paragraph. |  |
|  | 19 | Discuss limitations of the study, taking into account sources of potential bias or imprecision; discuss both direction and magnitude of any potential bias | Discussion, Limitations paragraph. Confounding by indication, non-randomised allocation, heterogeneous DXA equipment without cross-calibration, differential follow-up intervals, small sample size with the minimum detectable difference quantified, and absence of systematically collected fracture data. |  |
|  | 20 | Give a cautious overall interpretation of results considering objectives, limitations, multiplicity of analyses, results from similar studies, and other relevant evidence | Discussion and Conclusion. The apparent early advantage of teriparatide is interpreted as not independent of baseline bone density, and no strategy is recommended over another. |  |
|  | 21 | Discuss the generalizability (external validity) of the study results | Discussion and Limitations. All participants were identified at tertiary endocrine centers, and follow-up scheduling and duration of anabolic therapy were shaped by national reimbursement criteria. |  |
| Other information | 22 | Give the source of funding and the role of the funders for the present study and, if applicable, for the original study on which the present article is based | Funding statement. No financial support was received; the Turkish Society of Endocrinology and Metabolism supported the nationwide collaboration among participating centers. |  |

Adapted from von Elm E, Altman DG, Egger M, Pocock SJ, Gøtzsche PC, Vandenbroucke JP; STROBE Initiative. The Strengthening the Reporting of Observational Studies in Epidemiology (STROBE) statement: guidelines for reporting observational studies. Lancet. 2007;370:1453–7.

The checklist and its explanatory paper are available at <https://www.strobe-statement.org>

**Supplementary Table S3.** Dual-energy X-ray absorptiometry systems in use at the 18 participating centres

| DXA system | Centers, n |
| --- | --- |
| Hologic | 9 |
| GE Lunar | 8 |
| FUJIFILM FDX Visionary | 1 |
| Total | **18** |

Manufacturer information was obtained from all 18 participating centres. Model designations, software versions and centre-level calibration records were not available, and no cross-calibration study was performed; BMD values were not converted to a standardised scale. All densitometric measurements for a given woman were obtained at the centre at which she was followed, so that within-patient change — the basis of all comparisons reported here — is not affected by differences between manufacturers, whereas absolute BMD values and Z-scores pooled across centres should be interpreted with this limitation in mind.
